# Phenotypic and molecular characterization of antimicrobial resistant *Escherichia coli* from urinary tract infections in Port-Harcourt, Nigeria

**DOI:** 10.11604/pamj.2019.34.144.18182

**Published:** 2019-11-13

**Authors:** Adebola Onanuga, Jaspreet Mahindroo, Shreya Singh, Neelam Taneja

**Affiliations:** 1 Department of Pharmaceutical Microbiology and Biotechnology, Faculty of Pharmacy, Niger Delta University, Wilberforce Island, Bayelsa State, Nigeria; 2 Department of Medical Microbiology, Postgraduate Institute of Medical Education and Research, Chandigarh, India

**Keywords:** *Escherichia coli*, urinary tract infections, multi-drug resistance, Extended Spectrum Beta Lactamases, quinolone, gentamicin, resistance genes

## Abstract

**Introduction:**

Multidrug resistance among *Escherichia coli* causing Urinary Tract Infections (UTIs) is a major public health problem, threatening the effective treatment of UTIs. This study investigated the phenotypic and molecular characteristics of *E. coli* associated with UTIs in Port-Harcourt, Nigeria.

**Methods:**

Twenty-five non-duplicate isolates of *E. coli* from UTIs patients at the University of Port-Harcourt Teaching Hospital, Nigeria were identified using Matrix-Assisted Laser Desorption Ionization Time-of-Flight (MALDI-TOF) Mass Spectrometry. The antimicrobial susceptibility patterns were determined using Kirby-Bauer disc diffusion technique. Phenotypic expression of Extended Spectrum Beta Lactamases (ESBLs) and AmpC beta-lactamase were determined using standard laboratory methods and polymerase chain reaction (PCR) was used to detect ESBLs, AmpC, Quinolones and Aminoglycosides resistance genes.

**Results:**

The isolates exhibited high rates of resistance to co-trimoxazole (76%), nalidixic acid (68%), ciprofloxacin (60%), gentamicin (44%) and low resistance to cefotaxime (20%) but were fully susceptible to cefoperazone/sulbactam, amikacin, nitrofurantoin, colistin and carbapenems. Phenotypic expression of ESBLs was recorded in 6(24%) isolates while genotypic detection revealed the highest prevalence of *bla*TEM 22(88%), followed by *bla*CTX-M-15 16(64%), blaSHV 7(28%) and *bla*OXA-1 6(24%) while AmpC (*bla*CMY-2) gene was detected in 8(32%) isolates. Amongst the quinolone resistant isolates, *qnr* variants (*qnrB*, *qnrD* and *qnrS*) and *aac(6')-Ib* genes were detected in 7(28%) and 3(12%) isolates respectively while all gentamicin resistant isolates possessed the *aacC2* gene. The co-expression of *bla*CTX-M-15 with quinolones and aminoglycoside genes were 20% and 40% respectively. The prevalence of multiple drug resistance was 52%.

**Conclusion:**

A high proportion of the studied *E. coli* isolates co-expressed ESBLs, quinolones and aminoglycosides resistance genes which call for prompt antibiotic stewardship and preventive strategies to limit the spread of these genes.

## Introduction

*Escherichia coli* strains are common bacteria that inhabit human gastrointestinal tract, whilst they are often harmless commensals; they can cause multitude of infections such as urinary tract infections (UTIs), meningitis, diarrhoea and septicemia [1]. Their harmless strains can remain commensals as long as they do not acquire genetic elements encoding virulence factors which may eventually result in these diseases [2]. The alarming increase in the rate at which these strains acquire antibiotic resistance genes has limited therapeutic options especially for UTIs for which extensive use of antibiotics has been witnessed in both community and hospital settings [1, 3]. Extended Spectrum Beta Lactamases (ESBLs) expression among *E. coli* strains encodes resistance to oxyiminocephalosporins and many other important groups of antibiotics, thereby causing impediment to treatment of its infections [4, 5]. Also, the carbapenems which are the last resort in the effective treatment of severe ESBL-producing *E. coli* infections, have recently witnessed rise in resistance by *E. coli* strains that produced carbapenem-hydrolyzing enzymes [4, 6, 7]. Aminoglycosides have been an essential component of the antibiotic armory in the treatment of serious life threatening infections and UTIs caused by *E. coli*, but the increasing wind of antibiotic resistance across the globe has reduced their effectiveness, rendering some members of this class of antimicrobials virtually useless in certain *E. coli* infections [8]. The ineffectiveness of aminoglycosides has been attributed to the expression of aminoglycoside-modifying enzymes {nucleotidyltranferases (ANTs), phosphotransferases (APHs), or acetyltransferases (AACs)} which catalyze the modification of the 2-deoxystreptamine nucleus or the sugar moieties [9]. An increase in resistance to gentamicin has been reported amongst isolates of *E. coli* associated with UTIs in many parts of Nigeria and other African countries [10-12].

The advent of fluoroquinolones, the new generation of quinolones antimicrobial agents brought a ray of hope to the treatment of various infections caused by multi-drug resistant bacteria and became the drug of choice for the empiric therapy of most serious life threatening infections [1, 3]. However, the extensive use of these agents in clinical settings has made bacteria to develop resistance to them all over the world [3, 13]. Fluoroquinolones are one of the most widely used drugs in the treatment of UTIs but their frequent use in both community and hospital settings has led to a dramatic rise in resistance amongst *E. coli* causing UTIs [7, 12, 13]. Quinolones inhibit the DNA replication in *E. coli* strains by targeting the bacterial DNA gyrase (topoisomerase II) and topoisomerase IV (parC) enzymes but mutations in the specific domains of *gyrA, gyrB, parC* and *parE* can cause changes in single amino acid of either gyrase or topoisomerase IV leading to the bacterial resistance to quinolones [14]. High-level of fluoroquinolone resistance in *E. coli* strains has been attributed to multiple mutations in the quinolone-determining resistant regions (QRDR) of topoisomerase enzymes [1, 9]. Various community and hospital based studies from Nigeria and other African countries have reported a varying prevalence of phenotypic and genotypic ESBL producing enterobacteriaceae [15-19]. However, information on molecular characterization of *E. coli* isolates causing UTIs from Nigeria is sparse. Therefore, this study was carried out to investigate the molecular characteristics of drug resistance in *E. coli* isolated from patients with UTIs in Port Harcourt, Nigeria.

## Methods

**Bacterial strains:** a total of one hundred and forty urine samples obtained from patients of average age 29.6 years comprising of 60% females, presented to the Out-Patients Department of the University of Port Harcourt Teaching Hospital (UPTH), Port Harcourt, Nigeria in August 2015 with clinical symptoms of UTIs, were cultured on Cysteine Lactose Electron Deficient (CLED) medium before incubated at 37°C for 24 h for bacterial growth. The isolates of *E. coli* with a significant growth of ≥ 10^5^ cfu/ml were identified using conventional biochemical tests at the department’s laboratory in Nigeria and later confirmed using Matrix-Assisted Laser Desorption Ionization Time-of-Flight (MALDI-TOF) Mass Spectrometry, at the Department of Medical Microbiology, Postgraduate Institute of Medical Education and Research (PGIMER), Chandigarh, India. This study was approved by the Ethics and Research Committee of Niger Delta University, Wilberforce Island, Nigeria, before the commencement of sample collection.

**Antimicrobial susceptibility testing:** antimicrobial susceptibility testing of the confirmed *E. coli* isolates was performed on Mueller Hinton agar plates using modified Kirby-Bauer disc diffusion technique in accordance with the Clinical and Laboratory Standards Institute guidelines (CLSI, 2016) for amikacin (10 μg), gentamicin (10 μg), ciprofloxacin (5 μg), norfloxacin (10 μg), nalidixic acid (30 μg), nitrofurantoin (300 μg), co-trimoxazole (25 μg), cefoperazone (75 μg), cefoperazone/sulbactam (75/10 μg), tazobactam/piperacillin (110 μg), imipenem (10 μg), ertapenem (10 μg), meropenem (10 μg), colistin (10 μg), cefotaxime (30 μg) and cefotaxime/clavulanic acid (30/10 μg) (Becton Dickinson, USA). Minimum inhibitory concentrations (MICs) of gentamicin and ciprofloxacin were determined for all the strains using microbroth dilution method in accordance with CLSI guidelines [20]. *Escherichia coli* ATCC 25922 was used for quality control in both tests. Multi-drug resistance (MDR) in this study was defined as resistance of an isolate to at least one agent in three or more classes of antibiotics [21].

**Phenotypic detection of ESBLs:** screening for ESBLs production in all *E. coli* strains was done using combination disc diffusion method on Mueller Hinton (MH) agar plates. The single discs of Cefotaxime (30 μg) and combination discs of Cefotaxime/Clavulanic acid (30/10 μg) were placed on each of the isolate (at a turbidity of 0.5 McFarland standard) inoculated Mueller Hinton agar plate and incubated at 37°C for 24 hours for the detection of ESBL enzymes. The zone diameter around each of the two discs was measured and if the diameter around Cefotaxime/Clavulanic acid was 5 mm or more greater than the zone diameter around the single disc of Cefotaxime, the bacterial isolate is said to be an ESBL producing organism [20].

**Phenotypic detection of AmpC:** the ethylenediaminetetraacetic acid (EDTA) discs were prepared by applying 20 μl of a 1:1 mixture of sterile normal saline and 100 X Tris-EDTA solution to sterile discs (Himedia, India) and allowed to dry before being stored at 2 to 8°C. Each of the Mueller Hinton (MH) agar plates surface was then inoculated with a lawn of susceptible *E. coli* ATCC 25922 at a turbidity of 0.5 McFarland standard, according to the standard disc diffusion technique, the stored EDTA discs were immediately rehydrated with 20 μl of sterile saline prior to use before several colonies of each of the isolates were applied to an EDTA disc surface. A cefoxitin (30 μg) disc was placed on the surface of the MH agar and an EDTA disc was then placed almost touching the antibiotic disc with the inoculated disc surface touching (in contact with) the agar surface. The plates were then incubated inverted at 37°C for 24 hours, after which they were examined for either an indentation or a flattening of the zone of inhibition, indicating enzymatic inactivation of cefoxitin (positive result), or the absence of a distortion, indicating no significant inactivation of cefoxitin (negative result) as described by Black *et al.* [22].

**Preparation of DNA template for PCR amplification:** the DNA templates of each of the confirmed pure *E. coli* isolates were generated by dispensing most of the pure colonies of the overnight growth of each of the isolates on MaConkey agar into 100 μL 1X Tris-EDTA buffer, vortex mixed and boiled at 100°C for 10 minutes. Then transferred immediately to the freezer (-20°C) for 10 minutes, maintained at room temperature, vortex mixed and centrifuged at 10,000 rpm for 10 minutes. The resulting supernatant containing DNA of each of the isolates was collected, stored at 4°C and used as DNA template for PCR.

**Molecular detection of ESBLs encoding genes:** polymerase chain reaction (PCR) was performed for *bla*OXA-1, *bla*CMY-2, *bla*SHV, *bla*TEM and *bla*CTX-M-15 to detect the presence of extended spectrum beta lactamases encoding genes, as previously described by Peirano *et al*. [23] and Taneja *et al*. [24] using the primers in Table 1. The PCR amplification was carried out in a GeneAmp 9700 Thermal Cycler (Applied Biosystems, USA) using a 25 μl reaction mixture containing DNA template (1 μl), Taq buffer of 1.5 mM MgCl_2_ (2.5 μl), 0.4 mM dNTPs (1 μl) (Bangalore Genei, India), 0.5 μl each of forward and reverse primers (Sigma Aldrich, India), 0.5 μl of *Taq* polymerase (Bangalore Genei, India) and PCR grade water (19 μl), at initial denaturation at 94°C for 3 min; 40 cycles of 94°C for 30 sec, 55°C for 30 sec and 72°C for 1 min; and a final elongation step at 72°C for 7 min. The annealing temperature was 55°C for *bla*OXA-1 and *bla*TEM, 58°C for *bla*CMY-2, 59°C for *bla*SHV and 64°C for *bla*CTX-M-15 genes. The amplified PCR products were analyzed on a 1.5% w/v agarose gel stained with ethidium bromide (10 μg/ml) and electrophoresis was performed in 0.5X TBE buffer at 100 V for 60 minutes with a 100 bp DNA ladder as a molecular marker, and the gels were subsequently visualized in a gel documentation system (Alpha Innotech, AlphaImager 3400).

**Table 1:** Primers used for PCR amplification and sequencing

Gene Target	Primer sequence (5' → 3')	Amplicon size (bp)	Source
*bla*OXA-1	F - ATGAAAAACACAATACATATCAACTTCGC	820	Peirano *et al.*[23]
	R - GTGTGTTTAGAATGGTGATCGCATT		
*bla*CMY-2	F - AAATCGTTATGCTGCGCTCT	332	Taneja *et al.* [24]
	R - CCGATCCTAGCTCAAACAGC		
*bla*SHV	F - AGCCGCTTGAGCAAATTAAA	318	Taneja *et al.* [24]
	R - CGCTGTTATCGCTCATGGTA		
*bla*TEM	F - ATGAGTATTCAACATTTCCCG	859	Peirano *et al.*[23]
	R - ACCAATGCTTAATCAGTGAG		
*bla*CTX-M-15	F - CCAGAATCAGCGGCGCACGA	587	Taneja *et al.* [24]
	R - GCGCTTTGCGATGTGCAGCA		
*Par*C	F - AAACC TGTTCAGCGCCGCATT	395	Vila *et al.* [26]
	R - GTGGTGCCGTTAAG CAAA		
*gyr*A	F - TACACCGG TCAACATTGAGG	648	Chu *et al.*[25]; Roderova *et al.* [1]
	R - TTAATGATTGCCGCCGTC GG		
*qnr*B	F - GATCGTGAAAGCCAGAAAGG	469	Robicsek *et al*. [27]
	R - ACGATGCCTGGTAGTTGTCC		
*qnr*D	F - CGAGATCAATTTACGGGG	582	Robicsek *et al*. [27]
	R - AACAAGCTGAAGCGCCTG		
*qnr*S	F - TGGAAACCTACAATCATA	656	Robicsek *et al*. [27]
	R - TTAGTCAGGATAAACAAC		
*aac(6*′*)-Ib*	F - ATCTCATATCGTCGAGTG	376	This study
	R - CGCTTTCTCGTAGCATCG		
*aph*A2	F - GAACAAGATGGATTGCACGC	688	This study
	R - GCTCTTCAGCAATATCACGG		
*aad*A1	F - CATCATGAGGGAAGCGGTG	787	This study
	R - GACTACCTTGGTGATCTCG		
*aad*A2	F - GTACGGCTCCGCAGTGGATGGCGG	537	This study
	R - GCCCAGTCGGCAGCGACATCCTTC		
*aac*C2	F - CGGAAGGCAATAACGGAG	740	This study
	R - TCGAACAGGTAGCACTGAG		
*aac(3)*-IV	F - GTGTGCTGCTGGTCCACAGC	628	This study
	R - AGTTGACCCAGGGCTGTCGC		

**Amplification of the quinolone resistance determining regions (QRDRs):** the QRDRs of *gyr*A and *par*C genes were amplified in a representative batch of isolates as previously described by Roderova *et al.* [1] and Chu *et al.* [25] for *gyr*A gene and Vila *et al.* [26] for *par*C genes using the primers in Table 1. For each 25 μl reaction mixture containing a strain’s DNA template, the condition used for the amplification of *gyr*A was initial denaturation at 94°C for 3 min, 30 cycles of 92°C for 1 min; 64°C for 1 min; 74°C for 2 min and a final cycle of 74°C for 10 min. The condition for *par*C was 30 cycles at 94°C for 1 min, 55°C for 1 min, and 72°C for 1 min.

**Amplification of the plasmid mediated quinolone resistance (PMQRs):** the PMQRs genes for *qnr*B, *qnr*D, *qnr*S and *aac(6')-Ib* were amplified as previously described by Robicsek *et al.* [27] and Guessennd *et al*. [28] using the primers in Table 1. The conditions used for the amplification of each 25 μl reaction mixture containing DNA template for *qnr*B, *qnr*D and *qnr*S were 94°C for 45 s, 53°C for 45 s, and 72°C for 60 s, for 32 cycles, while that of *aac(6')-Ib* was 94°C for 45 s, 55°C for 45 s, and 72°C for 45 s for 34 cycles.

**Amplification of the aminoglycosides resistance genes:** the strains were screened for the presence of aminoglycosides resistance genes by amplifying the genes for *aph*A2, *aad*A1, *aad*A2, *aac*C2 and *aac*(3)-IV using the primers described in Table 1. The conditions used for the amplification of each 25 μl reaction mixture containing DNA template were at initial denaturation at 94°C for 2 min; 35 cycles of 94°C for 45 sec, 56°C for 45 sec and 72°C for 1 min; and a final elongation step at 72°C for 5 min. The annealing temperature was 56°C for *aad*A1 and *aac*C2, 58°C for *aph*A2, 64°C for *aac* (3)-IV and 72°C for *aad*A2 genes.

**Sequencing of QRDRs genes:** all amplified products of *gyr*A and *par*C genes from selected strains were sequenced to validate their identities. Both strands of the purified amplicons were sequenced with a Genetic Analyzer (ABI Prism 3200 sequencer; Applied Biosystems), using the same primers as used for PCR amplification. Nucleotide and deduced protein sequences were analysed and compared in BLAST of the National Center for Biotechnology Information (NCBI) database.

**Statistical analysis:** the groups differences were tested using the Chi-square test (or Fisher’s exact test when expected frequencies were too low), with the assumed level of statistical significance at a P-value of < 0.05. Data analysis was performed with SPSS version 15.0 for Windows (SPSS Inc, USA).

## Results

**Figure 1:**
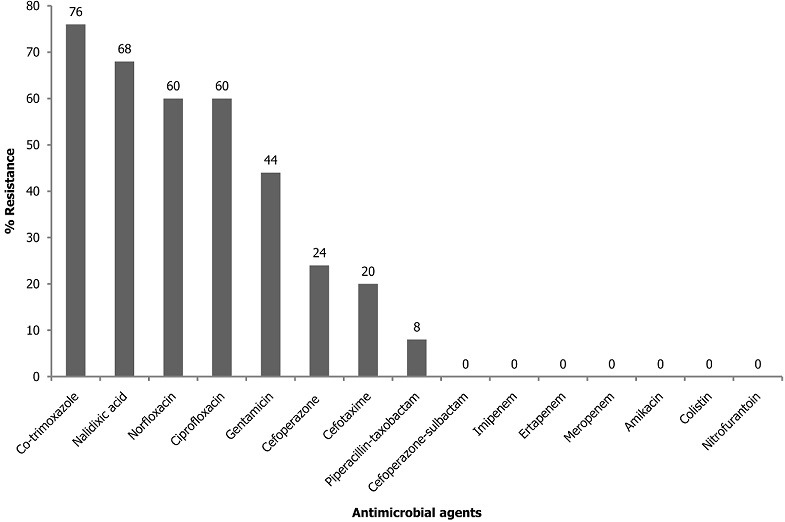
Antimicrobial resistance profile of urinary tract isolates of *Escherichia coli*

In total, only 25 non-duplicate *E. coli* isolates from patients with UTIs were identified. The antimicrobial resistance profile of the isolates revealed a high resistance (60 -76%) to co-trimoxazole (folate inhibitor) and quinolones-fluoroquinolones group, moderate resistance to gentamicin and low resistance to taxobactam/piperacillin and the cephalosporins. All isolates were susceptible to cefoperazone/sulbactam, amikacin, nitrofurantoin, colistin and carbapenems as described in Figure 1. The MICs determination of gentamicin and ciprofloxacin revealed that 12 (80%) isolates that were resistant to ciprofloxacin and 5 (45.4%) isolates resistant to gentamicin had a high level of resistance with MICs of 256-512 μg/ml. Three (12%) isolates had an MIC of 256 μg/ml for both gentamicin and ciprofloxacin. A total of 13 (52%) isolates exhibited multiple drug resistance (MDR). The MDR in this study was observed to be significantly associated with resistant isolates having MIC ≥ 128 μg/ml to gentamicin (p = 0.011) and to ciprofloxacin (p = 0.0001).

**Figure 2:**
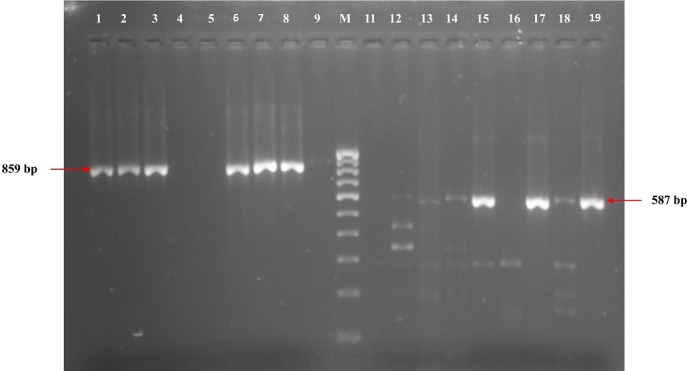
Representative gel for the detection of *blaTEM* and *blaCTXM-15* genes

A total of 6 (24%) isolates expressed ESBLs phenotypically while none expressed AmpC. The comparison of the antimicrobial resistance of the ESBL and non-ESBL producing isolates revealed significant differences only in cefoperazone (p = 0.015) and cefotaxime (p = 0.005) as showed in Table 2. The PCR screening revealed the presence of the following ESBLs encoding genes (Figure 2): *bla*OXA-1 (6, 24%), *bla*SHV (7, 28%), *bla*CTX-M-15 (16, 64%) and *bla*TEM (22, 88%). All the isolates that phenotypically expressed ESBLs had both *bla*CTX-M-15 and *bla*TEM. Additionally, 4 (66.7%) of them also had *bla*SHV gene whilst the other 2 (33.3%) also had *bla*OXA gene. The plasmid mediated AmpC (*pAmpC*) gene *bla*CMY-2 was detected in 8 (32%) of the isolates. A total of 7 (87.5%) of these *pAmpC* producing isolates had at least one of the screened ESBLs genes while all the screened ESBLs genes were detected in one isolate (Table 3). Amongst the MDR isolates, 10 (76.9%) had at least two ESBLs encoding genes while 3 (30%) isolates also had *pAmpC* and ESBLs encoding genes. The isolates that possessed *bla*OXA gene were significantly associated with MDR (p = 0.039) than those that possessed other screened ESBL genes. However, no significant association was observed between the isolates that possessed *pAmpC* gene (*bla*CMY-2) and exhibition of MDR (p = 0.411).

**Table 2:** Antimicrobial resistant pattern of ESBL and Non-ESBL producing *E. coli* isolates

Antimicrobial agents	Resistant isolates (%) n = 25	ESBL (%) n = 6	Non-ESBL (%) n = 19	p-value*
Cefoperazone	6 (30.8)	4 (66.7)	2 (10.5)	0.015**
Cefotaxime	5 (38.5)	4 (66.7)	1 (5.3)	0.005**
Taxobactam/Piperacillin	2 (15.4)	0	0	NA
Gentamicin	11 (76.9)	4 (66.7)	7 (36.8)	0.350
Co-trimoxazole	19 (100)	6 (100)	13 (68.4)	0.278
Nalidixic acid	17 (100)	4 (66.7)	13 (68.4)	1.000
Norfloxacin	15 (100)	4 (66.7)	11 (57.9)	1.000
Ciprofloxacin	15 (92.3)	4 (66.7)	11 (57.9)	1.000
MDR	13 (52.0)	4 (66.7)	9 (47.4)	0.645

* *P value* (from Fisher’s exact test) is the comparison of resistance of ESBL and non-ESBL producers

** = Statistically significant (*P* ≤ 0.05)

NA = Not applicable

**Table 3:** Results of phenotypic and molecular detection of resistance in strains of *E. coli* causing UTIs

Strain Code	Antibiogram Patterns	MIC (μg/ml)		Phenotypic		Genotypic		Other Resistance genes		ORDR	
		CIP	GEN	ESBLs	AmpC	pAmpC	ESBLs	PMQRs	AGS	*Par*C	*Gyr*A
P1	NA, NOR, CIP, GEN,	32	512	NEG	NEG	Negative	*bla*SHV, *bla*TEM, *bla*CTX-M-15	Negative	*aac*C2	ND	ND
P2	NA, NOR, CIP, GEN, COT	32	256	NEG	NEG	Negative	*bla*TEM	Negative	*aac*C2	ND	ND
P3	CFT, CFP, NA, NOR, CIP, GEN, COT	256	256	POS	NEG	Negative	*bla*SHV, *bla*TEM, *bla*CTX-M-15	Negative	*aac*C2	POS	POS
P6	NA, COT	0.25	0.5	NEG	NEG	Negative	*Bla*TEM, *bla*CTX-M-15	Negative	ND	POS	POS
P7	Fully susceptible	0.125	0.5	NEG	NEG	*bla*CMY-2	*bla*TEM, *bla*CTX-M-15	Negative	*aad*A2*, aac*C2	POS	POS
P8	NA, NOR, CIP,	256	1	NEG	NEG	*bla*CMY-2	*bla*TEM	Negative	ND	ND	ND
P9	TZP, NA, NOR, CIP, COT	512	2	NEG	NEG	*bla*CMY-2	*bla*OXA-1, *bla*TEM *bla*CTX-M-15	*qnr*D, *aac(6*′*)-Ib*	ND	POS	POS
P10	COT	0.125	1	NEG	NEG	*bla*CMY-2		Negative	*aad*A2	ND	ND
P11	CFT, CFP, NA, NOR, CIP, GEN, COT	512	32	POS	NEG	Negative	*bla*OXA-1, *bla*TEM *bla*CTX-M-15	*qnr*D, *aac(6*′*)-Ib*	*aac*C2, *aac*(3)-IV	POS	POS
P12	CFT, CFP, COT	1	1	POS	NEG	Negative	*bla*SHV, *bla*TEM, *bla*CTX-M-15	Negative	ND	ND	ND
P13	CFP, NA, COT	1	2	NEG	NEG	Negative	*bla*SHV, *bla*TEM	Negative	ND	POS	POS
P14	COT	0.125	1	POS	NEG	*bla*CMY-2	*bla*CTX-M-15	Negative	ND	ND	ND
P15	COT	0.125	2	NEG	NEG	Negative	*bla*TEM	Negative	ND	ND	ND
P16	NA, NOR, CIP	32	1	NEG	NEG	Negative	NEG	Negative	Negative	ND	ND
P19	CFT, CFP, TZP, NA, NOR, CIP, COT	512	4	NEG	NEG	Negative	*bla*OXA-1, *bla*TEM	*qnr*D, *qnr*S	ND	ND	ND
P20	Fully susceptible	1	1	NEG	NEG	Negative	*bla*TEM and *bla*CTX-M-15	Negative	ND	POS	POS
P21A	NA, NOR, CIP, GEN, COT	256	256	POS	NEG	Negative	*bla*SHV, *bla*TEM, *bla*CTX-M-15	*qnr*D, *qnr*S	*aad*A2*, aac*C2	ND	ND
P21B	NA, NOR, CIP, GEN, COT	256	128	NEG	NEG	Negative	*bla*TEM	*qnr*D, *qnr*S	*aad*A2*, aac*C2	ND	ND
P22	COT	0.125	1	NEG	NEG	*bla*CMY-2	*bla*TEM, *bla*CTX-M-15	Negative	ND	ND	ND
P23	NA, NOR, CIP, GEN, COT	256	64	NEG	NEG	*bla*CMY-2	*bla*TEM	Negative	*aac*C2	ND	ND
P24	NA, NOR, CIP, GEN, COT	256	128	NEG	NEG	Negative	*bla*TEM, *bla*CTX-M-15	Negative	*aad*A1, *aad*A2*, aac*C2	ND	ND
P25	NA, NOR, CIP, GEN, COT	256	128	NEG	NEG	Negative	*bla*SHV, *bla*TEM and *bla*CTX-M-15	*qnr*S	*aad*A1, *aad*A2*, aac*C2	ND	ND
P26	CFT, CFP, NA, NOR, CIP, GEN, COT	256	256	POS	NEG	*bla*CMY-2	*bla*OXA-1, *bla*SHV, *bla*TEM and *bla*CTX-M-15	*qnr*B, *aac(6*′*)-Ib*	*aad*A1, *aad*A2*, aac*C2	POS	POS
P28	NA, NOR, CIP, GEN, COT	256	128	NEG	NEG	Negative	*bla*OXA-1, *bla*TEM, *bla*CTX-M-15	Negative	*aad*A1, *aad*A2*, aac*C2	ND	ND
P29	Fully susceptible	1	2	NEG	NEG	Negative	*bla*TEM, *bla*CTX-M-15	Negative	*aac*C2	ND	ND

CFT, cefotaxime; CFP, cepoferazone; TZP, taxobactam/piperacillin; NA, nalidix acid; NOR, norfloxacin; CIP, ciprofloxacin; GEN, gentamicin; COT, co-trimoxazole; ND; not determined; AGS, aminoglycosides; POS, positive; NEG, negative

**Figure 3:**
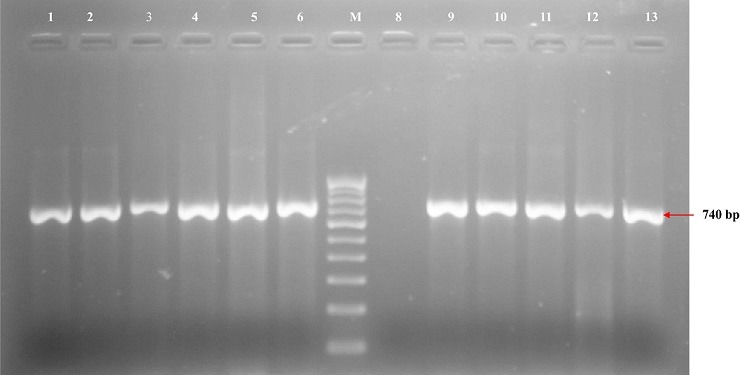
Representative gel for the detection of *aacC2* aminoglycoside gene

On PCR screening of *gyr*A and *par*C genes in eight randomly selected isolates, all were found to be positive. Three (37.5%) of these isolates also carried at least two of the screened β-lactamase and aminoglycosides encoding genes (Table 3). The screening for PMQRs genes revealed the presence of *qnr* variants (*qnrB*, *qnrD* and *qnrS*) and *aac(6')-Ib* genes in 7 (28%) and 3 (12%) of the isolates respectively. These were *qnrB* (n=1), *qnrD* (n=5) *qnrS* (n=4). Three isolates carried both *qnrD* and *qnrS* while another carried only *qnrS*. Two other isolates carrying *qnrD* also had *aac(6')-Ib* gene while the isolate carrying *qnrB* also had *aac(6')-Ib*. All these isolates had ciprofloxacin MICs of 256 - 512 μg/ml, and also carried at least two of the screened ESBLs encoding genes (Table 3). The prevalence of PMQRs genes among the MDR isolates was 7 (53.8%) and the MDR isolates were observed to significantly possess at least one of the ESBL genes and one of the PMQRs genes (p = 0.015). All the gentamicin resistant isolates possessed *aac*C2 gene with one or more of the other screened aminoglycosides genes except *aph*A2 gene (Figure 3). The isolates that possessed *aac*C2gene were significantly associated with MDR (p = 0.017) than those that possessed other screened aminoglycosides genes. Four (36.4%) of the isolates with MICs 128 - 512 μg/ml had *aad*A1, *aad*A2 and *aac*C2 genes with at least two of the screened β-lactamase encoding genes (Table 3). The MDR isolates were observed to significantly possess at least one of the ESBL genes and one of the aminoglycosides genes (p = 0.017).

## Discussion

*Escherichia coli* is one of the major causes of UTI affecting humans of all ages. The emergence of MDR *E. coli* strains and the progressive rise in antimicrobial resistance threatens the effective treatment of UTIs leading to increased morbidity, prolonged hospital stay, increase in the cost of treatment and disease related mortality [29]. Thus, early detection of the characteristics of antimicrobial resistance of this organism in a particular region will help to quickly adapt strategies that will reduce the potential misuse of antimicrobial agents and prevent the emergence and subsequent spread of such MDR isolate. The antimicrobial susceptibility test results of the UTI associated *E. coli* strains in this study revealed a very high resistance to co-trimoxazole. This observation which has been widely reported might be due to its extensive misuse as a first line drug of choice in the treatment of uncomplicated UTIs but its usefulness has now been hindered because of the high level of bacterial resistance [10, 11, 18, 30]. The fluoroquinolones and gentamicin which were among the most effective agents of choice in the empiric treatment of most bacterial infections in the last one decade were now observed in this study to be largely ineffective on these *E. coli* strains. This increasing level of resistance has also been reported in recent studies from other developing countries where there is no strict policy on the use of antibiotics in their communities [7, 12, 30].

The potentially high effectiveness of taxobactam/piperacillin, cefoperazone/sulbactam, amikacin, nitrofurantoin, colistin and all the tested carbapenems on the urinary *E. coli* strains in this study support previous findings [7, 12, 18, 30]. Hence, any of these agents or their combinations can be used in the empiric treatment of urinary tract infections. The effectiveness of nitrofurantoin, although one of the oldest UTIs drugs, is not unlikely to be attributed to its unpleasant side effects which has largely discouraged its frequent misuse and this supports previous findings [7, 11, 18]. The prevalence of multiple drug resistance among the studied isolates was 52% which is lower than the previously reported findings of 85-100% in various parts of Nigeria and other African countries [10-12, 15, 30]. The observed differences might be due to the differences in the screening techniques at various centres of study. However, the observed prevalence of MDR indicates that the isolates might have been inadvertently exposed to these antimicrobials either from the clinics or agricultural products since *E. coli* can easily get exposed to the drugs used in animal husbandry and food industry through ingestion. Hence, control use of antimicrobials in both agricultural and clinical settings could reduce the prevalence of MDR among uropathogens.

The phenotypic detection of ESBLs in this study was identified in 24% of the isolates using only cefotaxime and its clavulanic acid combination discs. However, it has been reported that the use of multiple agents including aztreonam, ceftazidime and cefotaxime in the screening for ESBLs enhances highest rates of detection among the isolates [7, 20]. This finding is higher than the previous studies in Amassoma, South-Southern Nigeria (9.6%) and in Libya (6.7%) [18, 31]. However, our finding is in concordance with the studies from Osogbo, South-Western Nigeria (25%), Cotonou of Benin (25%) and Iran (22.3%) [12, 32, 33]. Higher prevalences have also be reported in Benin, South-Southern Nigeria (44.4%), Jordan (54%) and Togo (93.4%) [7, 16, 30]. The prevalence of ESBLs producing UTIs *E. coli* is a worldwide problem which varies according to countries from regions to regions and it is significantly associated with the extensive use of broad spectrum antibiotics especially cephalosporins as revealed in this study (p = 0.015; 0.005). Notwithstanding, differences in the screening procedures of ESBLs estimation across the various study centres might also contribute to the observed varying values.

The molecular detection of ESBL resistance genes among the isolates in this study revealed that the *blaTEM* was the most predominant beta lactamase gene. This finding is similar to various reports across the globe [7, 12, 34]. This might be due to the presence of *blaTEM* on the highly mobile genetic elements which favours its spread among bacteria globally [34]. This study’s prevalence rate of *blaCTX-M-15* (64%), which was the next most predominant beta lactamases genes among the isolates, confirms its increasing prevalence among uropathogenic *E. coli* strains as reported in many studies in Africa and other parts of the world [17, 19, 35, 36]. This study reveals a strong association between phenotypic expression of ESBL and the presence of *blaSHV* (p = 0.032) or *blaCTX-M-15* (p = 0.045). This is because, either of these genes was significantly detected among isolates that expressed ESBL phenotypically than those non-ESBL isolates that did not. This assertion is however in agreement with the findings of Muhammad and Swedan [7] in Jordan. All the multi-drug resistant isolates in this study possessed at least one of the screened ESBLs genes while 76.9% of the MDR isolates possessed two or more ESBLs genes, which therefore suggests a possible association between the presence of ESBLs genes and the prevalence of multiple drug resistance among the isolates (p = 0.015). Thus, it may be proposed that ESBLs encoding genes in these isolates are a possible risk factor for multiple drug resistance.

AmpC β-lactamases (class C β-lactamases) are broadly distributed especially in regions where antibiotics are extensively misuse, and unlike ESBLs, they hydrolyse broad and extended spectrum cephalosporins and are resistant to β-lactamase inhibitors like clavulanic acid, taxobactam and sulbactam [37, 38]. Bacteria producing AmpC are characterized with higher degree infections causing patient morbidity and mortality, which necessitate the need to screen for these enzymes in these bacterial isolates [39]. None of the isolates expressed AmpC phenotypically however, previous studies in Nigeria have reported low prevalence of 2.8% in Kano [40] and 10.5% in Benin City [41] among *E. coli* isolates from clinical samples. Higher prevalence of phenotypic expression of AmpC (18.6%) was detected among UTIs *E. coli* isolates in Egyptian Hospitals [39]. The observed differences might be due to the different screening techniques in the various study centres.

The detection of six groups of plasmid mediated AmpC (*pAmpC*) genes using PCR analysis has been reported and they include ACC, DHA, CMY, EBC, FOX and MOX [42]. In this study, the presence of *blaCMY-2*, a common variant of CMY group of pAmpC genes was screened for among the *E. coli* isolates using its primers. A prevalence of 8 (32%) isolates was detected and 7 (87.5%) of them had at least one of the ESBLs genes while remaining one (12.5%) isolate had all the screened ESBLs genes. This indicates that the patients would have been previously exposed to cephalosporin therapy either through rational or irrational use of the antibiotics since extensive use of broad spectrum cephalosporins is among the factors that favour the increasing prevalence of AmpC production [43]. To the best of our knowledge, this is the first report of pAmpC (*blaCMY-2*) gene in *E. coli* isolates from Nigeria. However, the prevalence of acquired *pAmpCs* has been known to be usually influenced by geographical area and the period of study, hence its comparison between studies across the globe may be difficult [44]. Notwithstanding, several studies have reported *blaCMY-2* gene type of *pAmpCs* as a predominant determining factor for AmpC resistance in *E. coli* [39, 45]. The detection of *blaCMY-2* gene in this study confirms its worldwide distribution and its presence in different plasmids which suggest its ability to be mobilized as part of transferable fragment among bacteria species [46]. This then explains the increasing rate of spread of multiple drug resistance genes among bacterial species worldwide which tends to limit clinical therapeutic options. Susceptibility to carbapenems (imipenem, ertapenem and meropenem) was demonstrated in all the *pAmpC* and ESBL producing isolates in this study, making them a good treatment option for this type of MDR bacterial infections.

In this study, the horizontally acquired quinolone resistance genes PMQRs (*qnrB*, *qnrD* and *qnrS*) and *aac(6')-Ib*) were detected among isolates that had very high ciprofloxacin MICs of 256 -512 μg/ml and all the isolates also possessed at least two of the screened ESBLs genes. This finding is similar to the previous reports of Ogbolu *et al.* [32] in Nigeria and Namboodiri *et al.* [47] in Ghana. This observed results suggests the possibility of these resistance genes conferring very high ciprofloxacin resistance to the organism which may lead to clinical treatment failure with ciprofloxacin therapy while the co-existence of both PMQRs and ESBLs genes in these isolates may explain the reasons for their multidrug resistance capability (p = 0.015). This therefore supports the findings that the genes encoding ESBLs in *E. coli* are usually located on transferable plasmids that also carry other resistance determinants, such as *aac(6')-Ib-cr* gene which induces resistance to both aminoglycosides and fluoroquinolones, making it an important mechanism of dissemination of multidrug resistance among various bacterial species [12, 30, 32].

Increasing aminoglycoside resistance among *E. coli* associated diseases has been widely reported globally. This study revealed the presence of *aac*C2, *aad*A1 and *aad*A2 as the prominent genes among the isolates with high level of MICs 128-512μg/ml. These isolates were equally observed to possess two or more of the screened β-lactamase genes and 8 (88.9%) of them significantly possessed multidrug resistance capability (p = 0.017). This therefore suggests that rapid dissemination of multidrug resistance genes which is favoured by the co-existence of resistance genes on same mobile genetic elements tend to limit therapeutic options against bacterial infections [48]. Thus, there is need for strategies to control the rapid dissemination of these antibiotic resistance genes through implementation of strict guidelines for antimicrobial use in clinical practice, prevention of over the counter misuse of antibiotics and rational use of antibiotics in both human and agricultural activities.

## Conclusion

This study highlights the high prevalence of ESBLs, AmpC, fluoroquinolones and aminoglycosides resistance genes co-habiting MDR uropathogenic *E. coli* strains suggesting an increasing UTIs treatment failure with commonly used antibiotics. Thus, the need for molecular surveillance of MDR bacteria is crucial to the optimization of empiric treatment of UTIs. It is also extremely needful to strengthen strict compliance to antibiotic stewardship and enforcement of infection control practices in all our health institutions as a means of controlling the increasing spread of MDR bacteria.

### What is known about this topic

- ESBLs genes are commonly detected among antibiotic resistant *E. coli* strains associated with urinary tract infections, a disease that affects human of all ages;
- Treatment of UTIs in both hospital and community settings is increasingly threatened because of the increasing reports of MDR among uropathogens to the commonly available antibiotics;
- The genes encoding ESBLs in *E. coli* are located on transferable plasmids which also carry other aminoglycosides and fluoroquinolones genes resulting in rapid dissemination of MDR among bacterial species.

### What this study adds

- There is a strong association between phenotypic expression of ESBL and the detection of *blaSHV* and/or *blaCTX-M-15* gene in the bacteria in the study environment;
- The presence of ESBLs encoding genes in a bacterium is a possible risk factor for multidrug resistance since they are located on same plasmids that carry other resistance determinant genes;
- The detection of *blaCMY-2* gene, a prominent plasmid mediated AmpC gene among UTIs associated *E. coli* strains carrying other ESBLs genes is the first report in Nigeria.

## Competing interests

The authors declare no competing interests.
